# The epigenomic basis of common diseases

**DOI:** 10.1186/s13148-017-0313-y

**Published:** 2017-01-19

**Authors:** Euan J. Rodger, Aniruddha Chatterjee

**Affiliations:** 1 Department of Pathology, Dunedin School of Medicine, University of Otago, Hanover Street, P.O. Box 56, Dunedin, 9054 New Zealand; 2 Maurice Wilkins Centre for Molecular Biodiscovery, Level 2, 3A Symonds Street, Auckland, New Zealand

## Abstract

A report of the 6^th^ Epigenomics of Common Diseases Conference held at the Wellcome Genome Campus in Hinxton, Cambridge, UK, on 1–4 November 2016.

## Introduction

Epigenetic modification provides a stable mechanism by which cells with the same genotype can modulate their gene expression and exhibit different phenotypes. In the past two decades, excellent progress has been made to profile these modifications and our understanding of epigenetic marks has surpassed beyond the basic phenomenon of cellular heterogeneity. It is now established that epigenetic marks are altered in almost all common human diseases. The Epigenomics of Common Diseases meeting, 1–4 November 2016, provided an account of the progress made in this area and also indicated future areas that are yet to be addressed. Although disease focussed, several other aspects were discussed that are relevant to epigenetics as a field, including cellular heterogeneity, epigenomic association studies, emerging concepts in cancer epigenetics and new innovative techniques of broad application (such as single-cell analysis and epigenomic editing approaches). Here, we provide a brief report of some of the key ideas and themes discussed in this meeting and based on these, we speculate on future research directions.

### A needle in the haystack: insights from epigenome-wide association studies

Recent advances in high-throughput DNA analysis now enable researchers to examine epigenetic modifications across the genome, primarily DNA methylation marks, for association with numerous disease phenotypes. As such, epigenome-wide association studies (EWASs) have been fruitful in their findings but they also harbour their own unique challenges. EWASs provide an opportunity to investigate a large number of CpGs across large numbers of patients and controls to detect aberrant methylation signals at a population level [1]. Further, EWASs are an ideal platform to tap into large international resources and compare multiple datasets with custom-generated EWAS data. Examples of well-curated epigenomic datasets include the International Human Epigenome Consortium (IHEC), the EU-funded BLUEPRINT project, and the International Cancer Genome Consortium (ICGC). Although EWASs have been in use for several years now and thousands of datasets and several analytical tools have been reported, there is still a need to understand the potential biases and the nature of factors that could influence the interpretation of results. More sophisticated tools need to be developed to account for these factors. One observation to come out of this meeting, based on the commentary of multiple speakers, was that EWASs require robust analytical tools to detect epigenetic variants of interest and to adjust for confounding factors such as genetic effects and cellular heterogeneity.

Several speakers presented vignettes of many interesting EWAS findings, including Stephan Beck (University College London, UK) who shared some “new twists” in EWAS analytics and started off with a plea for authors and editors alike to ensure EWAS papers include CpG numbers. This oversight, he suggested, was akin to publishing a genome-wide association study without including rs numbers for single-nucleotide polymorphisms. Methylation of a CpG site and its relationship to the expression of a corresponding or nearby gene is very context specific. For example, the methylation of a particular gene promoter is likely to be very different to the methylation of the gene body. Methylation in either of these elements could have a different function for the specificity of the corresponding transcription [2]. Therefore, providing the methylation of a gene as a whole is almost meaningless if the context (i.e. the CpG number) is not described. In their analysis of >700 haemopoietic effector cell methylomes from monozygotic twins discordant for type 1 diabetes, Beck and colleagues were only able to identify a single significant differentially methylated CpG position (DMP). Undeterred, they shifted their focus from differences in mean methylation and used a novel approach to detect differentially variable positions (DVPs). The diabetes-associated DVPs were temporally stable and mapped onto regulatory circuits involved in cell cycle and immune cell metabolism. Similarly, they also applied this approach to methylomes from monozygotic twins discordant for rheumatoid arthritis and also to the discovery of DNA methylation and gene expression variability in normal blood cells. Substantial DNA methylation and gene expression variation in normal blood cells has also been reported in recent genome-wide studies [3–5]. Therefore, it is important to consider the normally occurring variation in methylomes for detecting differential methylation signals. Beck also presented several bioinformatic tools for EWAS interpretation such as eFORGE (http://eforge.cs.ucl.ac.uk), which identifies tissue or cell-type specific signals from Illumina 450K methylation array data [6], EpiDISH (freely available from https://github.com/sjczheng/EpiDISH), which can be used for epigenetic dissection of heterogeneity within a sample, and CORALINA (comprehensive guide RNA library generation through controlled nuclease activity), a universal method for generating guide RNA libraries for large-scale CRISPR-based genomic and epigenomic screening [7].

Bill Cookson (Imperial College London, UK) presented an EWAS of total serum immunoglobulin E (IgE), which is a central mediator in asthma and atopy, in peripheral blood leukocyte methylomes [8]. Of the 36 loci showing an association between methylation and IgE concentration, several loci were annotated to genes related specifically to eosinophil function (e.g. *IL5RA*), which is consistent with the presence of activated eosinophils in atopic subjects. Notably, a monoclonal antibody targeted to IL5RA is currently in phase 3 trials for the treatment of severe asthma. Cookson reiterated the point that cell heterogeneity and genetic factors need to be accounted for in EWASs. The WCGNA package (freely available from https://labs.genetics.ucla.edu/horvath/CoexpressionNetwork/Rpackages/WGCNA/) that was used to account for cellular heterogeneity in this study was suggested as an alternative to other more established methods that adjust for cell mixtures in the analysis of DNA methylation data.

Also from Imperial College London, UK, John Chambers reported findings from the EpiMigrant study, which investigated changes in DNA methylation patterns associated with high risk of type 2 diabetes in South Asians [9]. Methylation markers identified at five loci were found capable of predicting future onset of diabetes independently of other known risk factors. The genes associated with three of these loci (*TXNIP*, *ABCG1*, and *SREBF1*) have biological plausibility in the context of type 2 diabetes. In an EWAS investigating adiposity, methylation markers at 187 loci were found to be associated with body mass index (BMI) [10]. These loci were enriched for functional regulatory elements and gene promoters that mapped to biologically plausible pathways. However, Mendelian randomisation to test for causal relationships indicated that the changes in methylation were more likely to be a consequence rather than a cause of BMI.

### Epigenetics in cancer

The mechanisms involved in the hallmark properties of a cancer cell are well described and continue to be heavily investigated [11]. Until recently, cancer was often perceived as only a disease of the genome. However, it is now established that epigenetic changes are present in all human tumours and the fact that cancer is a disease of both the genome and epigenome is gaining recognition. The analysis of thousands of cancer genomes revealed that epigenomic regulator genes were often mutated in many cancers [12]. Epigenetic alterations have been shown to cooperate with genetic alterations to drive the cancer phenotype [13]. These changes can be used as biomarkers of disease state and the potentially reversible nature of epigenetic aberrations has been an alluring prospect for the field of epigenetic therapies.

In her talk on epigenetics of the cancer microenvironment, Susan Clark (Garvan Institute of Medical Research, Sydney, Australia) began by quoting Stephen Paget’s 1889 “soil and seed hypothesis”—“When a plant goes to seed, its seeds are carried in all directions; but they can only live and grow if they fall on congenial soil”. The focus of Clark’s investigation was on pro-tumorigenic cancer-associated fibroblasts (CAFs), which in vitro studies have shown, were not just transiently activated by signalling from tumour cells but they retain their phenotype even when tumour cell stimuli were removed. Whole-genome bisulfite sequencing (WGBS) and RNA-Seq were used on CAFs from prostate cancer and compared to matched non-malignant prostate fibroblasts (NPFs). This comparison showed a large number of discrete differentially methylated regions (DMRs), mainly hypomethylated, that were enriched at promoters and enhancers. A subset of DMRs shared methylation changes with tumour epithelial cells, suggesting convergent epigenetic programming. These robust biomarkers show promise for improved early prostate cancer diagnosis.

Peter Jones (Van Andel Research Institute, MI, USA) shared some compelling findings that support treating haematological and other cancers with a combination of epigenetic therapy, e.g. the DNA methylation inhibitor 5-aza-2′-deoxycytidine, and physiological levels of vitamin C. From in vitro studies of cancer cells, this combination treatment was shown to synergistically inhibit proliferation and increase apoptosis by enhanced demethylation of endogenous retroviruses (ERVs). The subsequent increased expression of ERVs stimulated immune signalling leading to apoptosis. Interestingly, it has been recently shown that treatment of ovarian cancer cell lines with a DNA methylation inhibitor triggered double-stranded RNA (dsRNA) sensing in the cytoplasm causing a type I interferon response and apoptosis. This response was associated with upregulation of hypermethylated ERV elements. They also observed a similar event in melanoma [14]. Taken together, these findings indicate that understanding the consequences of demethylation treatment in large genomic segments such as repeat elements (for example, ERVs) provide new opportunities to modulate the cancer epigenome and phenotype, resulting in better responses to new treatments such as immunotherapy. Further, Jones suggested that the enhanced ERV demethyltion effect was a result of both passive demethylation by 5-aza-2′-deoxycytidine and active demethylation by the TET family of enzymes, of which Vitamin C is a cofactor. Indeed, the ERV-induced response was blunted in TET2 knockout cells. As approximately 60% of cancer patients are significantly vitamin C deficient, these findings suggest that the response to epigenetic therapy in many patients could be improved by correcting for this deficiency.

Manel Esteller (Bellvitge Biomedical Research Institute, Barcelona, Spain) was able to showcase a large amount of impressive work to come out of his lab recently. These included a genome-wide DNA methylation analysis of cell lines derived from the primary melanoma tumour and matched lymph node metastasis from the same individual. From this analysis, Esteller and colleagues discovered that hypomethylation-associated reactivation of a cryptic 47kDa transcript of *TBC1D16* promotes melanoma growth and metastasis and in a clinical setting is associated with poor prognosis [15]. In an effort to explore regulatory regions outside classical coding and promoter regions, they have also used WGBS to identify cancer-associated DNA methylation aberrations in super-enhancers [16]—key regulatory regions associated with cell identity and function [17]. Of interest, in a later talk, Francois Spitz (Institut Pasteur, Paris) indicated that a super-enhancer can actually be a clustered collection of distinct specific enhancer molecules. Esteller also introduced a valuable resource that provides insight into pharmacogenomic interactions in cancer [18]. By mapping a large number of cancer-specific alterations from tumour tissues (mutations, copy number alterations, DNA methylation, and gene expression) onto well-annotated human cancer cell line pharmacogenomic datasets, it was found that the cell line data reliably recapitulated the cancer-specific alterations in tumours and could be used to predict drug sensitivity or resistance. Wrapping up, Esteller presented an analysis that used DNA methylation profiling of 2790 tumours with an unknown primary origin to establish a predictive classifier of the original primary tumour site. The predictive classifier was validated in a cohort of 7691 tumour samples with high specificity and selectivity [19].

Duncan Sproul (MRC Human Genetics Unit, University of Edinburgh, UK) described a work that used WGBS in oestrogen receptor-positive breast tumours to explore causes of widespread DNA hypomethylation in cancer. These tumours were specifically hypomethylated at large partially methylated domains (PMDs) that replicated in late S-phase and were associated with dysfunctional DNA methyltransferases. Loss of the de novo DNA methyltransferase DNMT3B resulted in preferential hypomethylation of PMDs, whereas loss of the maintenance DNA methyltransferase DNMT1 resulted in genome-wide hypomethylation. Furthermore, hypomethylation of PMDs was correlated with an increase in copy number variations that would contribute to chromosome instability in these tumours. Although the existence of PMDs in cancer cells and somatic cells has been described and analysed in several studies using different methods [20–23], their role in carcinogenesis is still unclear. One possibility is that the formation of PMDs could explain the potential mechanism of global hypomethylation observed in the cancer genome; however, reproducible functional studies need to be conducted in the future to establish the role of PMDs.

One technical aspect of genome-wide methylation profiling that was evident from both Susan Clark’s and Duncan Sproul’s talks was the power of using WGBS. Although 450K or 850K are very reproducible and are feasible platforms for large-scale EWAS, it was clear that large genomic methylation changes could escape detection of these array platforms, which have representative CpG probes for specified regions. For example, the use of WGBS enabled the detection of PMDs in breast cancer as discussed by Sproul. Susan Clark showed examples of some large regions showing differential methylation in prostate cancer, and this detection would not be possible with the existing array platforms. Sequencing-based genome-wide methods such as reduced representation bisulfite sequencing (RRBS) and WGBS are also more likely to detect methylation aberrations in regions distant from coding genes. Although the cost of WGBS is still substantially very high to be applied for large-scale studies at the moment, these techniques can be used for more niche questions on limited samples to gain biological insight for further exploration.

### Innovative epigenomic approaches

The field of epigenomics continues to rapidly expand, which is aided by researchers embracing or even developing new techniques and technologies to decipher unanswered questions. Two of the most exciting concepts to emerge recently are the use of single-cell profiling and targeted epigenetic genome editing using the CRISPR/Cas9 system.

Wolf Reik (Babraham Institute, Cambridge, UK) has used single-cell epigenomic profiling of embryonic stem cells (ESCs) to explore the relationship of epigenetic heterogeneity and cell fate decisions in mammalian development. DNA methylation analysis of naïve compared to primed ESCs showed the greatest methylation heterogeneity in enhancer elements. By modelling DNA methylation in primed ESCs, Reik suggested that oscillations of methylation and demethylation by the DNMT3 enzymes and the TET enzymes could explain the transcriptional heterogeneity observed in primed ESCs. Single-cell sequencing revealed global regulation of heterogeneity at gastrulation. The absence of “translational substructure” in the primed states indicated transcriptional noise was at a peak prior to major cell fate decisions. Reik concluded by briefly introducing a new single-cell method based on NOMe-Seq [24] that incorporates chromatin accessibility, methylation, and transcriptome analysis.

Continuing on in a similar vein, John Marioni (EMBL-EBI, Wellcome Genome Campus, UK) used single-cell RNA-Seq to explore cell fate decisions in early embryo development. Analysis of mouse embyros captured from early gastrulation to primitive erythrocyte formation identified ~2000 highly variable genes that were allocated to ten distinct clusters. Repeating this approach in Tal1 knockouts allowed sharper insight into whether this cell fate decision followed a step-wise restriction model [25]. In another study, Marioni and colleagues used single-cell sequencing of unstimulated CD4+ T cells in mice, which showed that T cell activation triggers a transcriptional switch from stochastic to tightly regulated gene expression. In older animals, the core activation program was expressed at a lower average upon immune stimulation. Further, ageing significantly perturbed the activation of the core program and increased variability between cells.

Emily Saunderson (Barts Cancer Institute, Queen Mary University of London, UK) from the laboratory of Gabriella Ficz presented a study that targeted a CRISPR/Cas9 DNMT 3A/3L fusion to a panel of genes in primary breast myoepithelial cells. The hypermethylation-associated repression of RASSF1 and CDKN2A resulted in increased cell proliferation. Outgrowing cells were not immortalized, but they escaped senescence. Interestingly, targeting the CpG islands of both CDKN2A gene products, p16 and p14, but not individually allowed the cells to escape senescence.

Daniel Ibrahim (Charité—Universitätsmedizin Berlin, Germany) and colleagues used CRISPR/Cas9 in mice to recapitulate genomic duplications at the *Sox9* locus that in humans caused female to male sex reversal when the duplications were contained within a non-coding chromatin-partitioning unit called a topologically associated domain (TAD). Capture Hi-C and 4C-seq analysis showed that intra-TAD duplications resulted in sex reversal and no overall change in TAD conformation. However, in contrast, inter-TAD duplications across TAD-boundaries were associated with a normal phenotype due to an insulation effect and new chromatin domains called neo-TADs were formed. However, when inter-TAD duplications included an adjacent potassium channel, *Kcnj2*, the duplicated gene was now located inside the neo-TAD and misexpressed by the *Sox9* regulatory landscape which caused a different disease, Cooks Syndrome [26].

Rieke Kempfer (Max Delbrück Center for Molecular Medicine, Berlin-Buch, Germany) from the Epigenetic Regulation and Chromatin Architecture laboratory of Ana Pombo introduced a novel cryosectioning-based method, genome architecture mapping (GAM), that can be used to explore relationships between 3D interactions and gene expression. This method has been applied to mouse ESCs, where specific domains have shown enrichment for interactions between distant enhancers and actively expressed genes. It is hoped that this method, which achieves 30 kbp resolution using as little as 400 cells, can be applied to a clinical setting in the future [27].

## Concluding remarks

In conclusion, this meeting covered many different concepts and new developments in the area of disease epigenetics. One aspect that generated some discussion was the correction for cellular composition in epignenetic profiling. Although sophisticated algorithms have been developed in the last few years to correct for methylation bias due to cell composition in the blood, it was clear from the discussion that the existing methods are far from perfect. It is important to note this potential bias as a result of mixed cell type, rather than assuming that the algorithms will provide the ideal results. This is particularly important as the blood continues to be the most widely used tissue for any epigenomic study (for example, in neurological diseases it is assumed to be a good surrogate for brain tissue, which is of course difficult to obtain). For other cell types (such as from the kidney, the skin), the methods are yet to be developed for cell composition correction. In summary, although hundreds of reference methylomes are now available, correcting for methylation biases due to cell composition continue to pose substantial challenges.

DNA methylation is generally perceived as a suppressive mechanism, and this perception was developed with the repeated observation of gene silencing due to promoter methylation. However, as we are now able to profile methylation at a genome-scale (i.e., beyond the promoter), it is becoming clear that the relationship of methylation with gene expression is more complex than the simple assumption of negative regulation of expression by promoter methylation. In fact, from several talks, it became clear that methylation changes alter distal regulatory elements, particularly enhancers, which could result in large changes to the transcriptional program. Talks from cancer epigenetics also highlighted the importance of investigating the relationship between DNA methylation and repeat elements. Currently, little is known about methylation and the regulation of distant elements and future research is likely to reveal more on the nature of these relationships.

It was also evident from the meeting that the development of new tools and approaches is an active area of research in this field. Especially with the release of many publicly available datasets, it is important to develop new tools that are accessible to biologists as eventually it has to be useful for them to address their research questions. From this meeting, it was evident that exciting research on understanding the basis of epigenomics of common disease will continue to take place in the future.

## Abbreviations

BMI: Body mass index
CAF: Cancer-associated fibroblast
DMP: Differentially methylated position
dsRNA: Double-stranded RNA
DVP: Differentially variable position
ERV: Endogenous retrovirus
ESC: Embryonic stem cell
EWAS: Epigenome-wide association study
ICGC: International cancer genome consortium
IHEC: International human epigenome consortium
NOMe-Seq: Nucleosome occupancy and methylation sequencing
NPF: Non-malignant prostate fibroblast
PMD: Partially methylated domain
RRBS: Reduced representation bisulfite sequencing
TAD: Topologically associated domain
WGBS: Whole-genome bisulfite sequencing
